# Application of Deep-Learning Algorithm Driven Intelligent Raman Spectroscopy Methodology to Quality Control in the Manufacturing Process of Guanxinning Tablets

**DOI:** 10.3390/molecules27206969

**Published:** 2022-10-17

**Authors:** Yi Tao, Jiaqi Bao, Qing Liu, Li Liu, Jieqiang Zhu

**Affiliations:** 1 College of Pharmaceutical Science, Zhejiang University of Technology, Hangzhou 310014, China; 2 Chiatai Qingchunbao Pharmaceutical Co., Ltd., Hangzhou 310023, China

**Keywords:** Guanxinning tablets, Raman spectroscopy, convolutional neural network, process analytical chemistry, extraction process monitoring, intelligent process analysis

## Abstract

Coupled with the convolutional neural network (CNN), an intelligent Raman spectroscopy methodology for rapid quantitative analysis of four pharmacodynamic substances and soluble solid in the manufacture process of Guanxinning tablets was established. Raman spectra of 330 real samples were collected by a portable Raman spectrometer. The contents of danshensu, ferulic acid, rosmarinic acid, and salvianolic acid B were determined with high-performance liquid chromatography-diode array detection (HPLC-DAD), while the content of soluble solid was determined by using an oven-drying method. In the establishing of the CNN calibration model, the spectral characteristic bands were screened out by a competitive adaptive reweighted sampling (CARS) algorithm. The performance of the CNN model is evaluated by root mean square error of calibration (RMSEC), root mean square error of cross-validation (RMSECV), root mean square error of prediction (RMSEP), coefficient of determination of calibration ($R_{c}^{2}$), coefficient of determination of cross-validation ($R_{cv}^{2}$), and coefficient of determination of validation ($R_{p}^{2}$). The $R_{p}^{2}$ values for soluble solid, salvianolic acid B, danshensu, ferulic acid, and rosmarinic acid are 0.9415, 0.9246, 0.8458, 0.8667, and 0.8491, respectively. The established model was used for the analysis of three batches of unknown samples from the manufacturing process of Guanxinning tablets. As the results show, Raman spectroscopy is faster and more convenient than that of conventional methods, which is helpful for the implementation of process analysis technology (PAT) in the manufacturing process of Guanxinning tablets.

## 1. Introduction

Quality control in the manufacturing process is an important issue to guarantee the quality of end-products of botanical drugs [1,2]. In 2004, the U.S. Food and Drug Administration (FDA) issued the process analytical technology (PAT) industry guide, pointing out that a variety of methods exist as an overall quality-control system for PAT, which are used to monitor key quality attributes of raw materials and intermediates in real time, and to provide a reliable guarantee for the quality of end-products [3]. Given this guideline, in-process analytical methods and techniques are required to provide real-time quality information on botanical drugs.

The Guanxinning tablet is a botanical drug which is clinically used for the treatment of coronary heart disease and angina pectoris. The raw materials of the Guanxinning tablet are *Salvia miltiorrhiza* and *Ligusticum chuanxiong hort*. The extract of *Salvia miltiorrhiza* has the pharmacological effects of anti-platelet aggregation, anti-thrombosis, promoting fibrin degradation, anti-myocardial ischemia, and antioxidant [4,5], whereas the extract of *Ligusticum chuanxiong hort* can dilate blood vessels, increase coronary flow, and improve microcirculation [6]. The conventional quality-control method used in the manufacture of Guanxinning tablets is high-performance liquid chromatography, which is used for determining the bioactive compounds, such as danshensu, ferulic acid, rosmarinic acid, and salvianolic acid B. The disadvantage of the HPLC method is that it is time-consuming and consumes large amounts of organic solvents. Meanwhile, the HPLC method is unsuitable for real-time monitoring. To address this issue, Raman spectroscopy serves as a suitable alternative.

Compared with conventional PAT techniques, Raman spectroscopy has the merits of being non-destructive, fast, and portable. Also, Raman spectroscopy has been widely used in molecular fingerprinting [7,8], pathogenic bacteria discrimination [9], tumor diagnosis [10], and so on. The Raman spectrum of water is very weak, so, Raman spectroscopy is suitable for detection uses in liquid samples [11,12]; however, the application of Raman spectroscopy to quality control in the production process of botanical drugs is still rare.

Since Raman spectroscopy alone is still unable to achieve real-time detection, it needs to be combined with machine-learning algorithms [13]. The convolutional neural network algorithm (CNN), which is a deep-learning algorithm, has received increasing attention. As compared to the traditional machine-learning algorithms, such as partial least squares regression (PLSR) and support vector machine regression (SVR), the deep-learning algorithm can simplify the spectral deconvolution process and improve the model accuracy, making it very suitable for processing big data in the manufacturing process.

In this paper, an intelligent deep-learning algorithm driven Raman spectroscopy analysis methodology was established for the rapid and non-destructive determination of the contents of four bioactive compounds and soluble solid in water extract samples of Guanxinning tablets, which were collected from the manufacturing process. The model performance of CNN was compared with that of PLSR and SVR.

## 2. Results and Discussion

### 2.1. Determination of Bioactive Ingredients by HPLC-DAD

**Figure 1:**
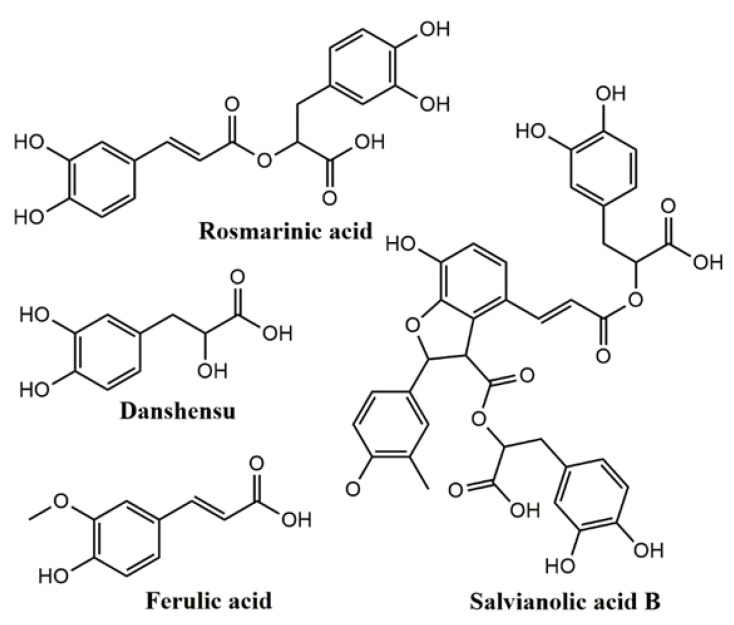
The chemical structures of the four investigated analytes.

**Figure 2:**
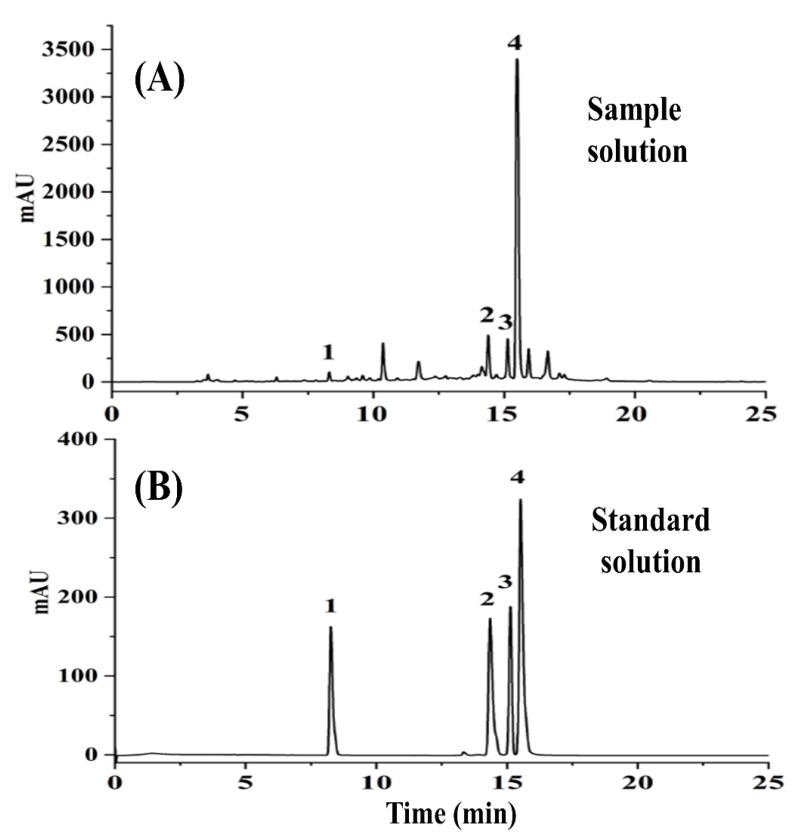
Representative HPLC chromatograms of sample solution (**A**) and standard solution (**B**).

A reliable HPLC-DAD method was established to determine the four bioactive ingredients. The chemical structures of the four analytes are shown in Figure 1. Danshensu, ferulic acid, rosmarinic acid, and salvianolic acid B have good UV-absorption characteristics at 288 nm. The representative chromatograms of real sample and mixed standard solutions are displayed in Figure 2. Peaks 1–4 represent danshensu, ferulic acid, rosmarinic acid, and salvianolic acid B.

335 real samples were analyzed by the HPLC-DAD method. All four bioactive compounds were baseline separated and could be accurately determined. Calibration curves, correlation coefficients, linearity ranges, and LOD and LOQ data are shown in Table S1. All four major compounds displayed good correlation coefficient values (r^2^) in the range of 0.9995–0.9997. The LODs and LOQs of the four major compounds were in the range from 0.2042 to 0.5313 μg/mL and from 0.6807 to 1.7709 μg/mL, respectively. The method was fully validated. The precision, repeatability, stability, and recovery of the method are shown in Table S2. The RSDs of intra-day and inter-day precisions of the method were determined to be in the range of 0.07–0.47% and 1.10–1.49%. The repeatability and stability of the method were determined as from 0.15% to 1.07% and from 0.11% to 1.03%. The overall recoveries ranged from 99.80% to 103.62%.

### 2.2. Determination of Soluble Solid by an Oven-Drying Method

The content of soluble solid is an important indicator of the water extracts. It is also necessary to establish a reliable reference method for determining soluble solid content. 335 samples were assayed by using the oven-drying method. The soluble solid of the water-extract samples ranged from 418.7 μg/mL to 4882.7 μg/mL.

### 2.3. Pretreatment of Raman Spectra

**Figure 3:**
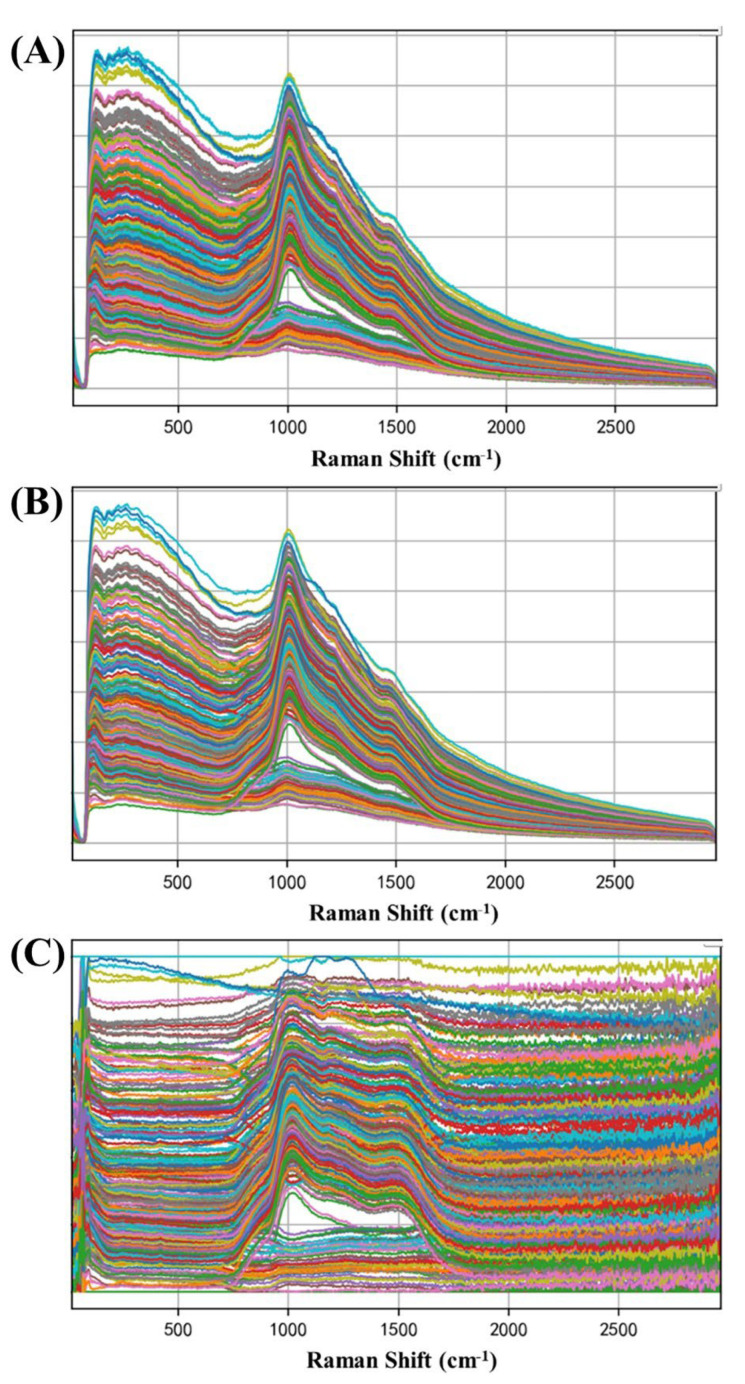
Raw Raman spectra (**A**) and spectra preprocessed by S−G (**B**) and Minmax (**C**) of water extract samples.

The raw Raman spectra of Guanxinning water extract are shown in Figure 3A. Savitzky–Golay (S–G) smoothing and Minmax linear regression were used (see Figure 3B,C). It can be seen in the raw spectrum that the major Raman peaks are 1000, 1250, and 1500 cm^−1^. The major Raman peaks were assigned by comparing with the literature [14,15]. The Raman peak at 1000 cm^−1^ can be ascribed to Ar ring stretching. The Raman peak at 1250 cm^−1^ can be ascribed to asymmetric stretching of the C-O-C bond. The Raman peak at 1500 cm^−1^ can be ascribed to C=C bond stretching.

### 2.4. Removal of Abnormal Spectra

**Figure 4:**
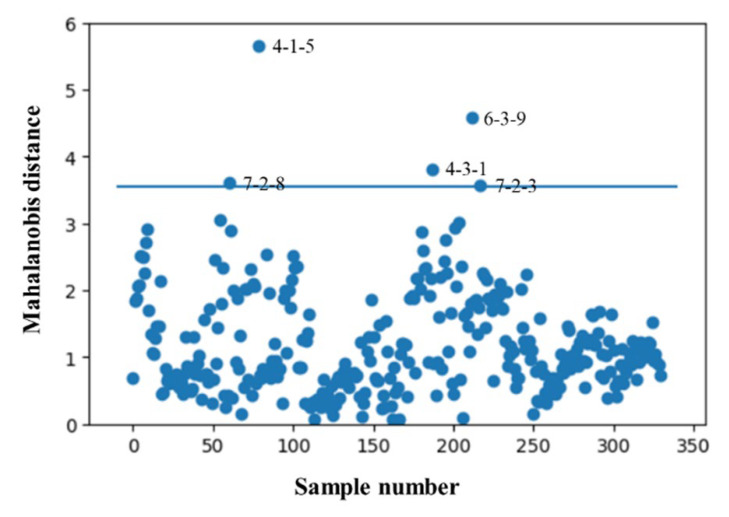
Mahalanobis distance distribution diagram of the samples.

The number of abnormal Raman spectra in 335 water-extract samples of Guanxinning was removed by using the Mahalanobis distance method. The Mahalanobis distance distribution of the samples is shown in Figure 4. Five abnormal samples (No. 4-1-5, No. 4-3-1, No. 6-3-9, No. 7-2-3, and No. 7-2-8) were identified and removed; therefore, the number of water-extract samples of Guanxinning for building a quantitative calibration model was 330.

### 2.5. Determination of Variable Selection Methods

Using the Kennard–Stone (K–S) algorithm, 330 real samples were divided into calibration set and validation set by 4:1. For each model, the calibration set consists of 264 samples, and the remaining 66 samples belong to the validation set. Table S3 lists the statistical values of the content of the four bioactive compounds in the calibration set and validation set. The calibration set covers a large range, which helps to build a stable and robust calibration model.

Four variable selection methods were used to select the characteristic bands of Raman spectra. CNN, PLSR, and SVR models were established, respectively. RMSEC, RMSEP, $R_{c}^{2},$ and $R_{p}^{2}$ were used to evaluate the performance of these models. Taking the PLSR model, for example, the calculation results are shown in Table S4. Among the four feature band selection algorithms, CARS shows the best prediction performance for danshensu, ferulic acid, rosmarinic acid, salvianolic acid B, and soluble solid with an $R_{p}^{2}$ at 0.6382, 0.8483, 0.9457, 0.8696, and 0.9282; thus, the CARS algorithm is adopted as the Raman spectral feature band selection method.

**Figure 5:**
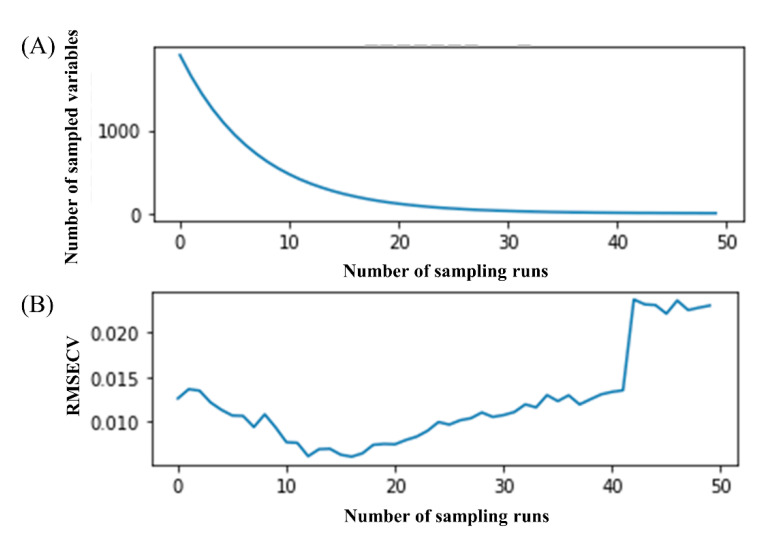
The process of selecting characteristic bands of Raman spectrum by CARS. (**A**) Number of sampled variables changed according to the number of sampling runs; (**B**) RMSECV values changed according to the number of sampling runs.

In the CARS algorithm, the Monte Carlo sampling rate was set to 0.8, and the sampling number was 50. Figure 5 represents a process diagram of CARS to extract variables. Taking rosmarinic acid as an example, when the number of times increases from 0 to 50, both the number of extractions changes (see Figure 5A) and the RMSECV values (see Figure 5B) change, but the change trends are obviously different. When the number of samples increases from 0 to 10, the number of selected variables decreases rapidly, which is the fast screening stage, and this process removes a lot of invalid information. When the number of samples is greater than 10, the number of variables shows a slow downward trend, which is the fine screening stage. When the number of samples is 16, the RMSECV value is the smallest, and the number of variables at this time is the optimal variable set.

### 2.6. Comparison of Different Calibration Models

**Figure 6:**
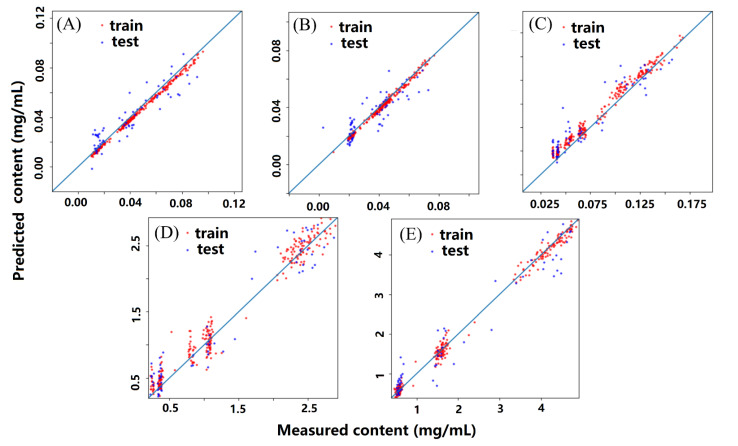
Correlation diagram of predicted values and measured values of the five attributes: (**A**) danshensu, (**B**) ferulic acid, (**C**) rosmarinic acid, (**D**) salvianolic acid B, and (**E**) soluble solid.

The performance parameters of the different calibration models established with the optimal band selecting method are listed in Table 1. According to the performance parameters, CNN, PLSR, and SVR were compared. It was worth mentioning that PSLR and SVR algorithms required preprocessing of the data. The calibration model of SPA-SVR showed the worst predictive ability, with an $R_{p}^{2}$ of −1.4600 and −0.0021. The calibration models of CARS-PLSR and CNN had modest predictive ability, with an $R_{p}^{2}$ of 0.6382, 0.8483, 0.9457, 0.8696, and 0.9282, and 0.8212, 0.7163, 0.8450, 0.8544, and 0.8554. The calibration model of CARS-CNN had the best predictive ability with an $R_{p}^{2}$ of 0.8458, 0.8667, 0.8491, 0.9246, and 0.9415. The RMSEP of the calibration model of CARS-CNN was the lowest. Correlation diagrams of predicted values and measured values of the five attributes of samples are shown in Figure 6.

### 2.7. Application to Three Batches of Unknown Samples

**Figure 7:**
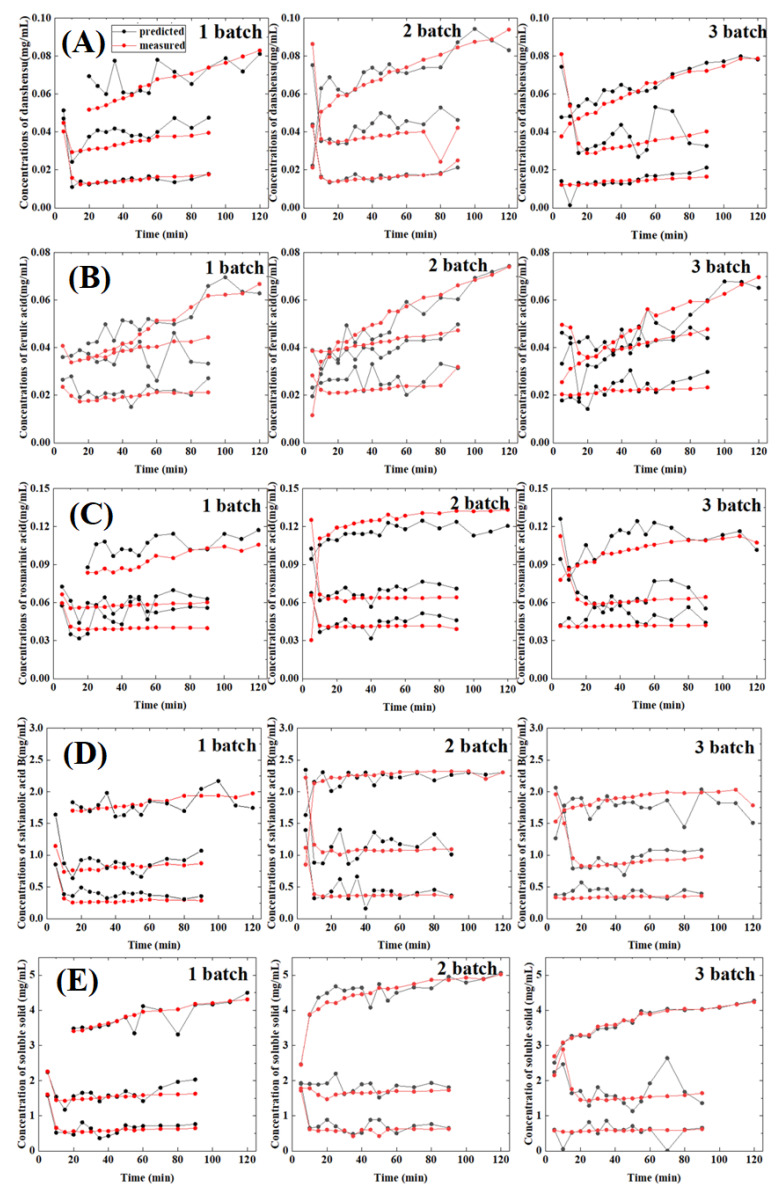
Contents of the five key attributes of three batches of unknown samples predicted with the established CARS-CNN model: (**A**) danshensu, (**B**) ferulic acid, (**C**) rosmarinic acid, (**D**) salvianolic acid B, and (**E**) soluble solid.

The established method is used for routine analysis in the production process, with which three batches of unknown samples of Guanxinning water extract were acquired by a portable Raman spectrometer. The Raman spectra of the unknown samples were corrected and inputted into the established CARS-CNN model. The contents of the four bioactive compounds and soluble solid were obtained at the same time. The three different batches of samples were analyzed with this model. Figure 7 shows the applications of Raman spectroscopy and CARS-CNN model to the unknown samples. The content of the main compounds is monitored and controlled during the production process through this method, allowing us to check whether the end-product meets the required standard and it further ensures the quality of the end-product. From Figure 7, we agreed that feasibility and superiority of CARS-CNN for the prediction of unknown samples is not apparent. The performance of deep learning highly depends on the size of the samples. The larger the sample size, the better the performance of the model will be. In our work, the sample size was 330, which is still too small for the CARS-CNN model. We believe the incorporation of more data of the samples into the model will unambiguously improve the performance and show the superiority of deep learning.

## 3. Materials and Methods

### 3.1. Sample Collection

**Figure 8:**
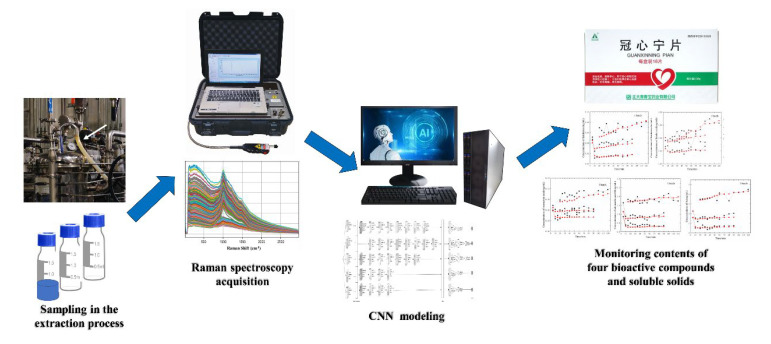
Schematic representation of the convolutional neural network enabled intelligent Raman spectroscopy methodology for monitoring the contents of four bioactive compounds and soluble solids in the water extracts of Guanxinning tablets.

Water-extract samples of Guanxinning tablets were collected from a Chinese medicine pharmaceutical factory (Zhengda Qingchunbao Pharmaceutical Co., Zhejiang, China) in Deqing. In the extraction process during the production of the Guanxinning tablets, reflux extraction was carried out three times. The extraction time of the first reflux process was 2 h. The extraction time of the second reflux process was 1.5 h. The extraction time of the third reflux process was 1.5 h. 10 mL samples were collected every 5 min for the first 1 h, and 10 mL samples were collected every 10 min for the next 1 h. When the crude drug enters the second and third reflux processes, 10 mL samples were collected every 5 min for the first 1 h, and 10 mL samples were collected every 10 min for the next 0.5 h. A total of seven batches of samples (335 samples) were collected from the Guanxinning water-extraction module. The port of extractor was spun off at certain time points and the water extract was poured into a small beaker. Then, the water extract was transferred to a centrifuge tube. The flow chart of this study is shown in Figure 8.

### 3.2. HPLC-DAD Analysis

In order to determine the concentration of four bioactive compounds in Guanxinning water extract, a high-performance liquid chromatography method was established. The water extract was centrifuged at 13,000 rpm for 10 min. Then, the supernatant was sent for analysis under the following chromatographic conditions. An Agilent 1260 high-performance liquid chromatography system (Agilent Technologies, Santa Clara, CA, USA) was used, including a quaternary pump, a sample vial injector, a column oven, and a diode array detector (DAD). The column was Hanbon Sci & Tech Hedera ODS-2 (4.6 × 250 mm, 5 μm), and the mobile phases consisted of (A) 0.1% HCOOH-H_2_O (*v*/*v*) and (B) acetonitrile. The gradient elution procedure was as follows: initial 95% (A); 0–12 min, 5–38% (B); 12–20 min, 38–48% (B); 20–35 min, 48–100% (B). The re-equilibration duration between single runs was 6 min. The column temperature was 36 °C and the flow rate was 0.8 mL/min. The detection wavelength of danshensu, ferulic acid, rosmarinic acid, and salvianolic acid B was 288 nm. LODs and LOQs were determined by using diluted standard solution when the signal-to-noise ratios (S/N) of the standard substances were about 3 and 10, respectively. Variations were expressed by relative standard deviations (RSD).

### 3.3. Oven-Drying Method

In order to determine the content of soluble solid in the Guanxinning water extract, an oven-drying method was adopted. Guanxinning water extract was centrifuged at 2500 rpm for 10 min. Then, about 3 mL of the supernatant was placed in a flat weighing bottle, evaporated to dryness in a water bath, and then placed in a 105 °C oven for 6 h. Finally, the bottle was taken out, placed in a desiccator to cool for 1 h, and weighed. The soluble solid content was calculated according to Formula (1), where *Sc* is the soluble solid content of the extract, *W* is the quality of the extract, *W*_2_ is the total mass of the sample and weighing bottle after drying, and *W*_1_ is the mass of the weighing bottle.

(1) $$S_{c}=\frac{W_{2}-W_{1}}{W}\times 100\%$$

### 3.4. Raman Spectra Acquisition

The Raman spectrum was collected by a Rapid OLRaman-2 portable Raman spectrometer equipped with a Raman fiber probe, a CCD detector, and a laser emitter (power 400 mW, wavelength 785 nm). It is a dispersive (with a grating) type of instrument. The acquisition parameters are as follows: wavenumber range 176–3500 cm^−1^, resolution 2.83 cm^−1^, acquisition time 500 ms, and samples were collected three times each. The Raman spectrometer was controlled by a compatible flat panel, and the “Pharmaceutical” software (Version 1.0) was used for data acquisition.

### 3.5. Removal of Abnormal Samples

In addition to the sample information, the data collected by the Raman spectrometer also include abnormal spectra that may have been generated due to errors of instrument, method, environment, or manual operation during the collection process. In order to obtain a reliable, accurate, and stable quantitative model, it is necessary to identify and remove abnormal spectra before modeling. In order to eliminate the interference of abnormal spectra, Mahalanobis distance method was used.

### 3.6. Feature Band Filtering

The Raman signal shift of the Raman spectrometer is 176–3500 cm^−1^. In order to best utilize the effective spectrum, the optimal characteristic bands should be screened out during the calibration process. To screen out the optimal spectral bands, competitive adaptive reweighted sampling (CARS), Uninformative Variable Elimination (UVE), Successive Projections Algorithm (SPA), and Synergy Interval Partial Least Square (siPLS) toolbox were used [16]. The performances of different screening algorithms were compared, and the best feature band screening algorithm was selected.

### 3.7. Determination of Variable Selection Methods

**Figure 9:**
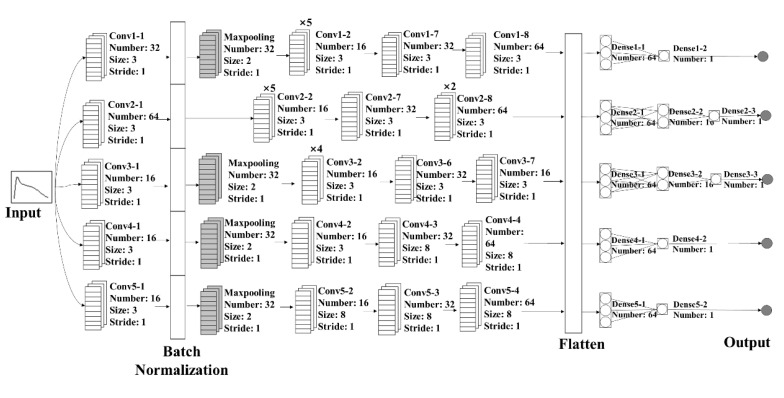
Architectures of the established CNN model.

The algorithms used for building the calibration models were PLSR, SVR, and CNN. The principle and application of these algorithms were well documented in the references [17,18,19]. The architecture of CNN model is shown in Figure 9.

The construction detail of the CNN model is as follows. First, a convolution layer was created. The parameters of the convolution layer were as follows: 32 filters, the filter window size was 3 × 3, the scanning window moved with a step size of 1 each time, and the rectified linear units (ReLU) activation function was applied. Second, a batch-normalization layer was created. Third, a maximum pooling layer was created. The number of filters in the pooling layer was the same as that of the convolution layer 1. The filter window size was 2 × 2, and the scanning window moved with a step size of 1 each time. There was no maxpooling layer for ferulic acid. Fourth, four convolutional layers with parameters setting to 16, 3, and 1 were created successively. The ReLU activation function was applied. After that, a convolution layer with parameters setting to 32, 3, and 1 was created and the ReLU activation function was applied. Then, a convolution layer with parameters setting to 64, 3, and 1 was created and the ReLU activation function was applied. Finally, a flattened layer was created. Then, two fully-connected layers were created for danshensu, salvianolic acid B, and soluble solid, whereas three fully-connected layers were created for ferulic acid and rosmarinic acid. The number of output neurons is 1, and the linear activation function was applied. The mean squared error loss function was chosen and Adam (lr = le × −4) was used as the optimizer. The specified batch size was 50. The number of iterations was 200. If the loss was not improved after 40 iterations, Keras would stop training.

The root mean square error of calibration (RMSEC), root mean square error of cross-validation (RMSECV), root mean square error of prediction (RMSEP), correlation coefficient of calibration ($R_{c}^{2}$), correlation coefficient of cross-validation ($R_{cv}^{2}$), and correlation coefficient of validation ($R_{p}^{2}$) were used to evaluate the performances of the above models. The detailed calculation formulas of the above parameters can be found in the literature [20].

## 4. Conclusions

An intelligent Raman spectroscopy methodology for the simultaneous determination of danshensu, ferulic acid, rosmarinic acid, salvianolic acid B, and soluble solid in water extract of Guanxinning was established. The calibration model has been validated with satisfactory $R_{p}^{2}$ values. The method has been successfully applied to the monitoring of the contents of pharmacodynamic substances and soluble solid in water extracts of Guanxinning tablets, which improves the efficiency of quality control, and may replace the cumbersome reference method. The model needs to be updated to ensure robustness for long-term use in industrial manufacturing. To the best of our knowledge, this study is the first to report the application of Raman spectroscopy in the analysis of pharmacodynamic substances and soluble solid in the manufacturing process of Guanxinning tablets. The proposed method is also expected to be useful for the implementation of process analytical techniques in the manufacturing of other botanical drugs.

## Figures and Tables

**Table 1:** Comparisons between performance parameters of four quantitative calibration models established with different regression algorithms.

Algorithms	Objectives	Calibration		Cross-Validation		Prediction	
		$R_{c}^{2}$	RMSEC	$R_{cv}^{2}$	RMSECV	$R_{p}^{2}$	RMSEP
SPA-SVR	Danshensu	−1.3462	0.0205	−1.6672	0.0176	−1.4600	0.0265
	Ferulic acid	−4.6801	0.0145	−5.2301	0.0423	−0.0021	0.0158
	Rosmarinic acid	0.5132	0.0424	0.4102	0.0644	0.3652	0.0448
	Salvianolic acid B	0.8718	0.2929	0.7189	0.2034	0.8100	0.3580
	Soluble solid	0.8185	0.6091	0.7033	0.7651	0.7189	0.7647
CARS-PLSR	Danshensu	0.9979	0.0011	0.9223	0.0031	**0.6382**	**0.0163**
	Ferulic acid	0.9843	0.0045	0.9456	0.0025	**0.8483**	**0.0057**
	Rosmarinic acid	0.9904	0.1539	0.9754	0.1634	**0.9457**	**0.0092**
	Salvianolic acid B	0.9958	0.0584	0.9192	0.1345	**0.8696**	**0.3749**
	Soluble solid	0.9904	0.1539	0.9312	0.2745	**0.9282**	**0.4512**
CNN	Danshensu	0.8694	0.0086	0.8423	0.0035	0.8212	0.0096
	Ferulic acid	0.8893	0.1197	0.7356	0.0768	0.7163	0.0213
	Rosmarinic acid	0.9285	0.0098	0.8749	0.0167	0.8450	0.0143
	Salvianolic acid B	0.8906	0.2988	0.8876	0.3758	0.8544	0.3371
	Soluble solid	0.8857	0.5389	0.8831	0.5775	0.8554	0.5812
CARS-CNN	Danshensu	0.9893	0.0024	0.9423	0.0051	**0.8458**	**0.0094**
	Ferulic acid	0.9875	0.0016	0.9335	0.0035	**0.8667**	**0.0084**
	Rosmarinic acid	0.9551	0.0078	0.9464	0.0079	**0.8491**	**0.0145**
	Salvianolic acid B	0.9943	0.1953	0.9213	0.1886	**0.9246**	**0.2528**
	Soluble solid	0.9526	0.1197	0.9384	0.3188	**0.9415**	**0.3861**

## Data Availability

The data and algorithm can be found at https://github.com/zjuttaoyi/-/tree/CNN.

## Supplementary Materials

The following supporting information can be downloaded at: https://www.mdpi.com/article/10.3390/molecules27206969/s1. Table S1. Calibration curves, correlation coefficients, linearity ranges, LOD and LOQ data of the four bioactive compounds. Table S2. Precision, repeatability, stability, and recovery of the four bioactive compounds. Table S3. The contents range of bioactive ingredients and soluble solid in training sets and test sets. Table S4. The performance parameters of the PLSR models established with different characteristic bands selecting methods.

## Sample Availability

Samples of the compounds are not available from authors.

## Abbreviations

CNN: convolutional neural network; CARS: competitive adaptive reweighted sampling; RMSEC: root mean square error of calibration; RMSECV: root mean square error of cross-validation; RMSEP: root mean square error of prediction; *R_c_*^2^: coefficient of determination of calibration; *R_cv_*^2^: coefficient of determination of cross-validation; *R_p_*^2^: coefficient of determination of validation; PAT: process analysis technology; DAD: diode array detector; UVE: Uninformative Variable Elimination; SPA: Successive Projections Algorithm; siPLS: Synergy Interval Partial Least Square; LV: latent variable; PRESS: predicted residual sum squared; K–S: Kennard–Stone; S–G: Savitzky–Golay; and ReLU: Rectified linear units.
