# Supplementary material for: Application of Deep-Learning Algorithm Driven Intelligent Raman Spectroscopy Methodology to Quality Control in the Manufacturing Process of Guanxinning Tablets
Source: Molecules. 2022 Oct 17;27(20):6969. doi: 10.3390/molecules27206969 (PMC9609342; doi:10.3390/molecules27206969)
Supplement: Supplementary file 1 [file molecules-27-06969-s001.zip › molecules-1965642-supplementary.pdf]

Table S1. Calibration curves, correlation coefficients, linearity ranges, LOD and LOQ data of the four bioactive compounds

Analytes	Calibration curves	r^2	Linear range (mg/mL)	LOD (μg/mL)	LOQ (μg/mL)
Danshensu	y=5933.2x+35.528	0.9997	0.0011841-0.9800	0.3552	1.1841
Ferulic acid	y=49767x-42.111	0.9997	0.0006807-0.3467	0.2042	0.6807
Rosmarinic acid	y=22842x-647	0.9995	0.0009837-1.0000	0.2951	0.9837
Salvianolic acid B	y=11620x+110.66	0.9995	0.0017709-3.2800	0.5313	1.7709

Table S2. Precision, repeatability, stability, and recovery of the four bioactive compounds

Analytes	Precision (RSD, %, n=6)		Repeatability	Stability	Recovery (%, mean/RSD, n=3)		
	Intra-day	Inter-day	(RSD, %, n=6)	(RSD, %, n = 6)	Low	Medium	High
Danshensu	0.07	1.24	0.15	0.14	103.16(0.32)	103.44 (0.43)	103.62(0.22)
Ferulic acid	0.47	1.49	0.76	0.11	99.80(0.60)	101.18(0.53)	100.71(0.47)
Rosmarinic acid	0.44	1.41	0.47	1.03	102.29(0.82)	102.01(0.77)	101.56(0.99)
Salvianolic acid B	0.23	1.10	1.07	0.64	101.61(1.28)	102.94(1.56)	102.47(1.68)

Table S3. The contents ranges of bioactive ingredients and soluble solid in training sets and test sets

Bioactive ingredients	Training sets (μg/mL)			Test sets (μg/mL)		
	min	max	mean	min	max	mean
Danshensu	10.6	95.9	50.6	12.5	81.6	47.2
Ferulic acid	19.2	76.7	48.1	21.6	63.5	43.5
Rosmarinic acid	36.9	167.7	98.3	37.0	161.7	89.4
Salvianolic acid B	223.4	2894.1	1551.1	225.9	2878.6	1662.3
Soluble solid	418.7	4882.7	2341.9	456.8	4846.6	2565.7

Table S4. The performance parameters of the PLSR models established with different characteristic bands selecting methods

**Method**	**Analysis**	**RMSEC**	R_c^2	**RMSEP**	R_P^2	**Selected wavelengths**
SPA	Danshensu	0.0105	0.7550	0.0110	0.7801	10
	Ferulic acid	0.0093	0.3746	0.0100	0.4021	10
	Rosmarinic acid	0.0137	0.8354	0.0333	0.8752	10
	Salvianolic acid B	0.3769	0.7879	0.3954	0.8096	10
	Soluble solid	0.6599	0.7890	0.7357	0.7528	10
siPLS	Danshensu	0.0064	0.9209	0.0123	0.7033	50
	Ferulic acid	0.0059	0.8089	0.0117	0.1848	50
	Rosmarinic acid	0.0095	0.9261	0.0152	0.8475	50
	Salvianolic acid B	0.2563	0.9119	0.3966	0.8241	50
	Soluble solid	0.4018	0.9309	0.6846	0.8471	50
UVE	Danshensu	0.0001	0.9999	0.0161	0.7000	7
	Ferulic acid	0.0001	0.9999	0.0089	0.6649	7
	Rosmarinic acid	0.0045	0.9844	0.0161	0.8406	7
	Salvianolic acid B	0.2518	0.9145	0.3307	0.8476	7
	Soluble solid	0.2809	0.9653	0.5517	0.8495	7
CARS	Danshensu	0.0011	**0.9979**	0.0163	**0.6382**	15
	Ferulic acid	0.0025	**0.9713**	0.0057	**0.8483**	17
	Rosmarinic acid	0.0045	**0.9843**	0.0092	**0.9457**	19
	Salvianolic acid B	0.0584	**0.9958**	0.3749	**0.8696**	16
	Soluble solid	0.1539	**0.9904**	0.4512	**0.9282**	15
